# The Focinator - a new open-source tool for high-throughput foci evaluation of DNA damage

**DOI:** 10.1186/s13014-015-0453-1

**Published:** 2015-08-04

**Authors:** Sebastian Oeck, Nathalie M. Malewicz, Sebastian Hurst, Justine Rudner, Verena Jendrossek

**Affiliations:** Institute of Cell Biology (Cancer Research), University of Duisburg-Essen, Medical School, Virchowstrasse 173, 45122 Essen, Germany

**Keywords:** Open-source tool, Foci count, Ionizing radiation, DNA repair foci, γ-H2.AX, ImageJ, Table sheet export, Automated analysis, Batch-mode, Colocalization

## Abstract

**Background:**

The quantitative analysis of foci plays an important role in many cell biological methods such as counting of colonies or cells, organelles or vesicles, or the number of protein complexes. In radiation biology and molecular radiation oncology, DNA damage and DNA repair kinetics upon ionizing radiation (IR) are evaluated by counting protein clusters or accumulations of phosphorylated proteins recruited to DNA damage sites. Consistency in counting and interpretation of foci remains challenging. Many current software solutions describe instructions for time-consuming and error-prone manual analysis, provide incomplete algorithms for analysis or are expensive. Therefore, we aimed to develop a tool for costless, automated, quantitative and qualitative analysis of foci.

**Methods:**

For this purpose we integrated a user-friendly interface into ImageJ and selected parameters to allow automated selection of regions of interest (ROIs) depending on their size and circularity. We added different export options and a batch analysis. The use of the Focinator was tested by analyzing γ-H2.AX foci in murine prostate adenocarcinoma cells (TRAMP-C1) at different time points after IR with 0.5 to 3 Gray (Gy). Additionally, measurements were performed by users with different backgrounds and experience.

**Results:**

The Focinator turned out to be an easily adjustable tool for automation of foci counting. It significantly reduced the analysis time of radiation-induced DNA-damage foci. Furthermore, different user groups were able to achieve a similar counting velocity. Importantly, there was no difference in nuclei detection between the Focinator and ImageJ alone.

**Conclusions:**

The Focinator is a costless, user-friendly tool for fast high-throughput evaluation of DNA repair foci. The macro allows improved foci evaluation regarding accuracy, reproducibility and analysis speed compared to manual analysis. As innovative option, the macro offers a combination of multichannel evaluation including colocalization analysis and the possibility to run all analyses in a batch mode.

**Electronic supplementary material:**

The online version of this article (doi:10.1186/s13014-015-0453-1) contains supplementary material, which is available to authorized users.

## Background

Radiotherapy (RT) is a mainstay in modern cancer treatment. To evaluate the efficacy of IR alone or in combination with chemotherapy or drugs inducing DNA damage and targeting DNA repair, radiation biologists usually count fluorescence-labeled protein-foci in the nucleus using fluorescence microscopy. For this purpose the proteins of interest or their specific phosphorylated isoform are visualized by immunofluorescence using protein-specific (e.g. p53 binding protein 1 (53BP1)) or phospho-protein-specific (e.g. phospho-histone 2.AX (γ-H2.AX)) antibodies directly linked to a fluorophore or detected by using a secondary fluorophore-labeled antibody. Another possibility is to fuse the proteins of interest with fluorescent proteins, such as green fluorescence protein GFP [1]. This method takes advantage of the fact that many repair proteins and repair associated proteins, such as γ-H2.AX, 53BP1 and RAD51, accumulate and co-localize at the site of DNA damage [2–8]. To evaluate formation and processing of these DNA damage foci, a reliable and accurate image analysis is required.

Due to the wide use of methods retrieving images of foci and cells, multiple evaluation procedures have been developed. However, the programs currently available for counting and analysis of nuclei are often based on manual analysis. Several publications showed that manual counting of foci is time consuming, frequently inaccurate and subjected to investigator-related bias. Conversely, automated computer-based foci analysis is considered to yield better sensitivity, comparability and consistency of the data [9–13]. However, some current software solutions for automated analysis are unsatisfactory as they provide limited algorithms, are stand-alone tools or are simply expensive [13, 14]. For example, Böcker *et al.* developed a software based on a cost-intensive program, ImageProPlus (Media Cybernetics Inc., US) [12]. Another commercially available package is IMARIS (Bitplane AG) [13, 15]. Moreover, not all existing tools support the complete range of file formats commonly used for image acquisition [16]. The FociCounter, a freely available, non-customizable stand-alone tool, does not support all formats, for example files used by Zeiss (CZI and ZVI) and by Leica (LIF). Moreover, the FociCounter only allows manual selection of cells [17]. However, integration of automated cell selection and a batch mode performing automated analysis of various pictures would result in desirable time-saving steps for data analysis. TRI2 and CellProfiler are stand-alone tools written with the programming language Python [18–20]. One disadvantage of stand-alone tools can be the lack of updates by an established platform. In contrast, the platform of ImageJ offers support, frequent updates and the possibility to change the source code or to link it with additional programming tools [9–13, 21, 22]. ImageJ-based solutions have already been described by several authors and institutions, but these solutions frequently provide incomplete algorithms or macros not suited for immediate use [23, 24]. For example, Cai and colleagues published the source code for an ImageJ macro without interface, like a menu and buttons [25], and Du and colleagues developed a tool for foci picking without batch mode and automated foci selection [26]. The FindFoci plugin for ImageJ supports self-learning parameters but does not support multi-channel analysis [10]. Thus, there was a demand for the development of easy-to-use, customizable and reliable software solutions with an intuitive interface combined with an automated open-source platform, like ImageJ [13]. To overcome these limitations, we have developed an automated, adjustable and user friendly macro based on ImageJ named “Focinator” for quantitative and qualitative analysis of nuclei, γ-H2.AX foci and other biological foci with the possibility of easy data export and processing. In addition, we integrated an option for multi-channel analysis, e.g. 53BP1 foci and γ-H2.AX foci in one image file and implemented the option for colocalization studies. This option enables the determination of absolute numbers and the percentages of colocalized foci. We used ImageJ as an established platform, as it is an image processing software that is routinely used by many investigators to analyze western blots, fluorescence cell images [13, 27], immunohistochemical probes [28], DNA double strand break repair [29], cell size [30] and to quantify soft tissue in tomography images [21] or wound healing [31]. We adapted the Focinator based on algorithms published by the Light Microscopy Core Facility -Duke University and Duke University Medical Center by adding additional setting preferences [24, 25]. To further facilitate data analysis, a program for automated analysis and data export into a spreadsheet was integrated.

## Materials and methods

### Chemicals, antibodies and drugs

Antibodies linked with Alexa Fluor 647 against γ-H2.AX protein were obtained from Becton Dickinson (Heidelberg, Germany). Hoechst 33342 from Invitrogen (Eugene, USA) and DAKO Fluorescent mounting medium from Dako North America Inc. (Carpinteria, USA) were used. All other chemicals were purchased from Sigma-Aldrich (Deisenhofen, Germany) if not otherwise specified.

### Cell culture and irradiation

TRAMP-C1 murine prostatic adenocarcinoma cells (p53−/−, androgen-independent) were purchased from the ATCC (Bethesda, Maryland, USA). Cells were cultured in Dulbecco’s Modified Eagle Medium (DMEM) (Life Technologies, Germany) supplemented with 10 % (v/v) fetal calf serum (FCS; Biochrom, Berlin, Germany) and maintained in a humidified incubator at 37 °C and 5 % CO_2_ (C200, Labotect Incubator, Goettingen, Germany). Cells were irradiated using an X-RAD 320 X-Ray Biological Irradiator with a MIR-324 X-ray tube (Precision X-Ray Inc., North Branford, USA). Cell number and viability was quantified by counting cells using CASY cell counter (Innovatis, Reutlingen, Germany).

### γ-H2.AX immunofluorescence

Cells were irradiated with 3 Gy and fixed and permeabilized (3 % para-Formaldehyde (PFA) and 0.2 % Triton X-100 in PBS buffer; 15 min; room temperature) at different time points (30 min, 1, 2, 4, 6, 8 and 24 h) after irradiation. After washing, cells were blocked overnight with 2 % goat serum in PBS buffer. Staining with the Alexa Fluor 647-conjugated anti-γ-H2.AX antibody was performed for one hour at a 1:75 dilution in blocking buffer. Samples were washed three times with PBS and stained for 30 min in the dark with 0.2 % (w/v) Hoechst 33342 in PBS. Samples were again washed three times with PBS, mounted with DAKO mounting medium and stored at 4 °C in the dark. Single layer fluorescence images were taken with a Zeiss AxioCam MRm (1388 × 1040 pixels) mounted at a Zeiss Axio Observer Z1 fluorescence microscope with Plan-Apochromat 63x/1.40 Oil M27 lens, 49 DAPI filter, 78 HE ms CFP/YFP filter (γ-H2.AX AF-647 detection) and “ApoTome” transmission grid (High Grid: PH/VH with 5 phase images) (Carl Zeiss, Goettingen, Germany). Images were taken with exposure times of 500 ms for the DAPI channel and 1500 ms for the Alexa Fluor 647 antibody. The pictures were saved as 16-bit Zeiss Vision Image ZVI files with no further editing.

### Software and programming

The macro “Focinator” was programmed as a macro for automated quantitative and qualitative analysis of foci with the open-source software ImageJ, a public domain Java image processing program developed at the National Institutes of Health (NIH) [14, 32]. ImageJ is designed with an open architecture and provides extensibility via Java plugins and automation with macros. Custom-built tools can be developed to solve image processing or analysis problems. [21, 22]. ImageJ is available for Windows, Mac OS, Mac OS X and Linux. It has its own ImageJ macro language that is able to control ImageJ procedures and the automation of action series including variables and user-defined functions. The macro, instructions and support are obtainable at http://www.focinator.oeck.de.

In addition, a tool for batch mode and import of data from foci-count and ROI analysis was developed: the batch mode was programmed using R, a free software environment for programming [33]. The R-script allows automated opening of images, foci evaluation and the direct export of foci data in a Microsoft Excel spreadsheet. Excel spreadsheets enable further statistical analysis as well as easy export into other statistical software.

### Foci analysis methods

For evaluations of foci counting, the respective groups counted γ-H2.AX foci formed in TRAMP-C1 cells at different time points after exposure to 3 Gy. Additionally, one experiment was performed with 0.5 to 3 Gy. In total 24,858 nuclei in 3361 images were counted. Two trained investigators performed manual foci-counting using a standard manual counter and two trained investigators analyzed foci with ImageJ. Three different user groups (in total 6 investigators) tested the Focinator’s user-friendliness: two programmers of the Focinator with prior knowledge of ImageJ and image processing software, two biologists with basic knowledge of image processing in a scientific context and two users without scientific background or prior knowledge of image processing. All investigators used the same workstations. The investigators did not receive any prior training in this software, but had to read the software’s instruction manual (Additional file 1: Supplement). The images were fully blinded before analysis; therefore investigators had no information about exposure details, dose, time points or type of analyzed cells. Manual counting, analysis with ImageJ and the Focinator were performed independently. Results from manual counting were not available for the investigators performing computational counting.

The software-based analysis for accuracy, comparability, validity and velocity was done by the primary developers of the Focinator (SO and NMM). Consequently, these people had prior knowledge of the image processing software.

### Statistical analysis

Data represent mean values of at least 3 independent experiments ± standard deviation (SD). Data analysis was performed by two-way ANOVA test with Bonferroni two pair comparison post-test and determination coefficient calculation using Prism5TM software (Graph pad Inc., La Jolla, USA). P values ≤ 0.05 were considered as significant.

## Results and discussion

### The Focinator

**Fig. 1:**
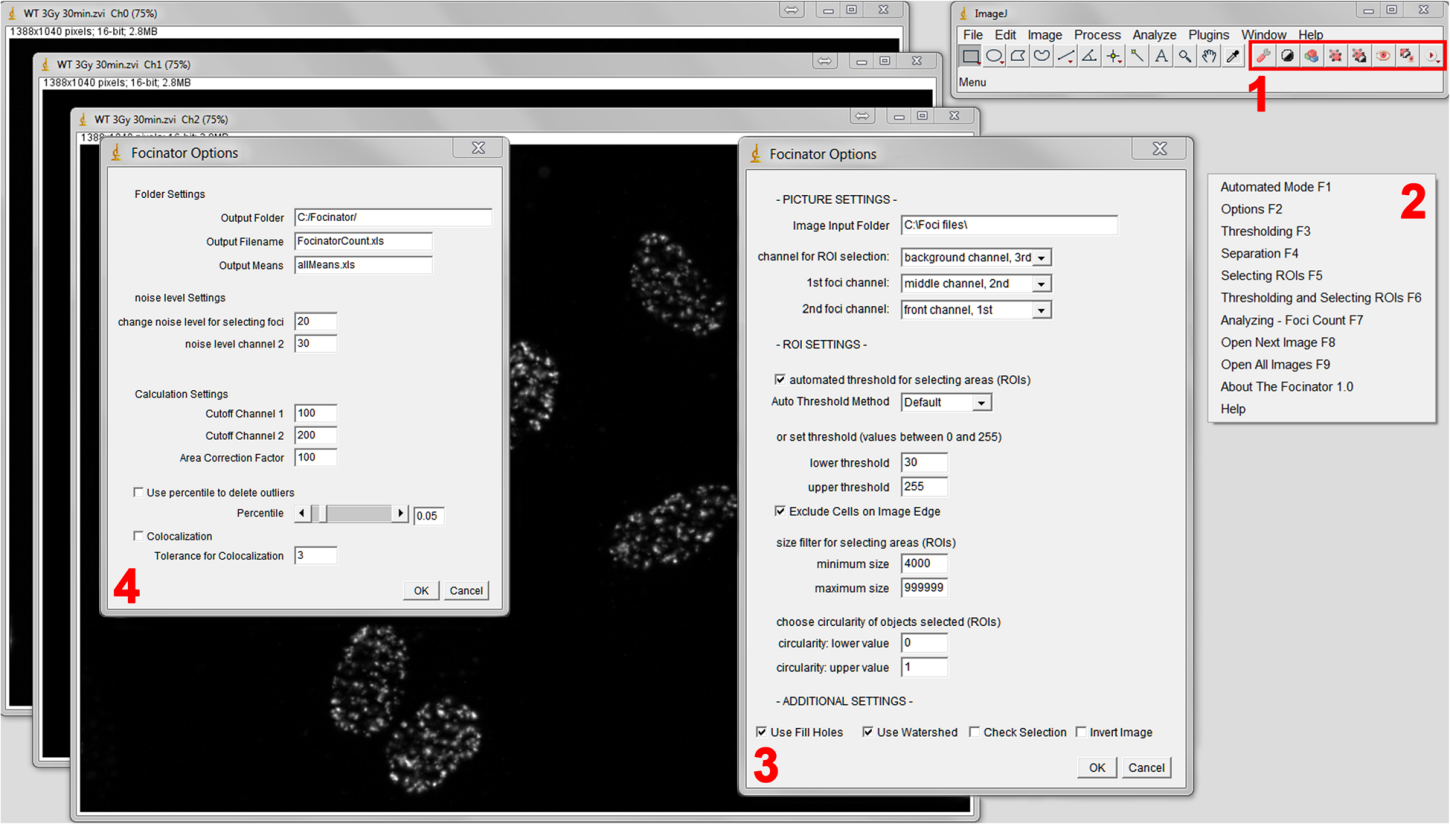
The ImageJ-based interface of the Focinator offers options to adapt the evaluation parameters to distinct image characteristics. Figure 1 shows ImageJ with the Focinator macro installed as start-up macro after opening a multi-channel image. This microscope image with the file format ZVI 16-bit includes three fluorescence channels. The main window of the Focinator is implemented into the ImageJ window. It consists of a menu (**2**), buttons (**1**) and Focinator Options (**3** and **4**). The Focinator Options windows offer several preferences for the user to adapt the macro’s behavior to individual requirements. Picture Settings: First step is to tell the macro, the input folder and if there is a multi-channel image or more single pictures will be opened. In the second step you choose in which channel the foci have to be counted and where the ROIs should be selected. In our example, the γ-H2.AX foci are in channel number 2 (*on top after opening the image*). The macro will use the setting “1st foci channel = front channel” for all pictures automatically. If no second foci channel is used the setting should be changed to “inactive”. ROI Settings (**3**): Depending on image quality, size and magnification, it is recommended to set the threshold and the size filters for ROIs. Alternatively, the choice of automated thresholding is possible. It is possible to exclude objects that are partially outside of the image. If there are objects to exclude because they are not circular enough or too small, it is possible to exclude them via circularity filters or size filters. “Use fill holes” should be activated, if the ROI selection left holes in the cells. Overlapping ROIs (cells, nuclei) might be separated by choosing “watershed”. Regarding the batch mode “check selection” offers the possibility of stopping during the selection process. “Invert images” should be checked when working with images with light background. For the automated batch (**4**) mode, output directories need to be chosen to save the results. An important step of evaluation is to choose the right noise level. Noise level values can be set independently in multi-channel analysis to exclude background artifacts. By defining the cut off, foci with intensities below a certain value are deleted, which excludes background noise. The value for area correction is dependent on the mean size of the analyzed nuclei. The factor corrects the foci number divided by the individual area of each nucleus. The usage of the percentile option enables the user to delete the outliers, such as cells with false γ-H2.AX foci induced by replication. Colocalization analyses are also possible. This option compares the localization of two foci in two different channels with a selectable tolerance

**Fig. 2:**
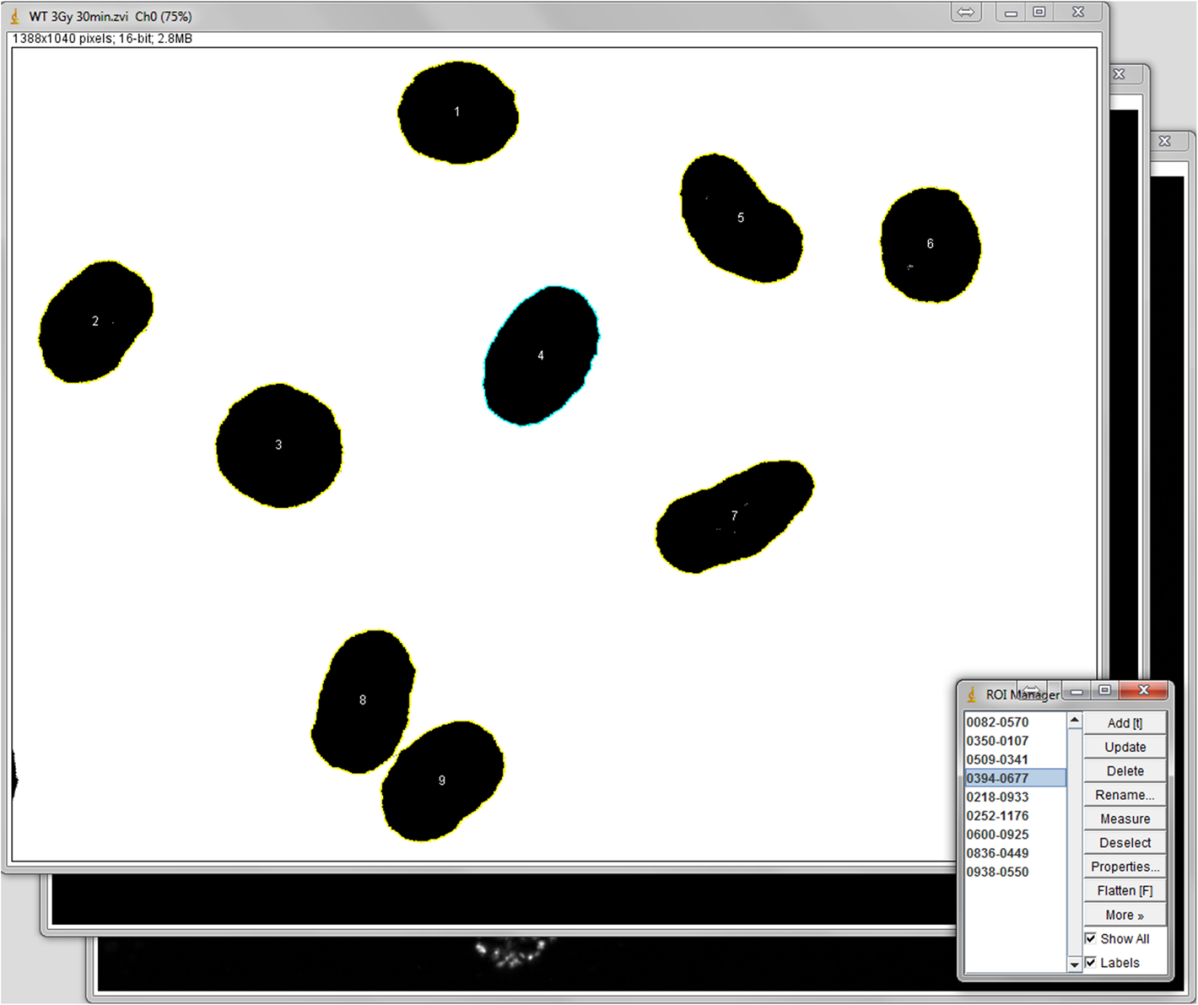
The macro automates the setting of the threshold and the contains an automated ROI selection. Figure 2 shows the calculation process frozen at the point of completed ROI selection. The ROI selection is necessary for the measurement of ROI area, intensity information (mean, maximum and minimum) and the foci count of each ROI (e.g. nucleus). Adjusting the threshold is the first step of ROI selection. The ROIs are marked by signal intensity-triggered selection of the areas. This selection and ROI marking is based on ImageJ “Create Selection” algorithm with options including filters for edge ROI exclusion, minimum and maximum size, watershed for overlapping objects and consideration of circularity

**Fig. 3:**
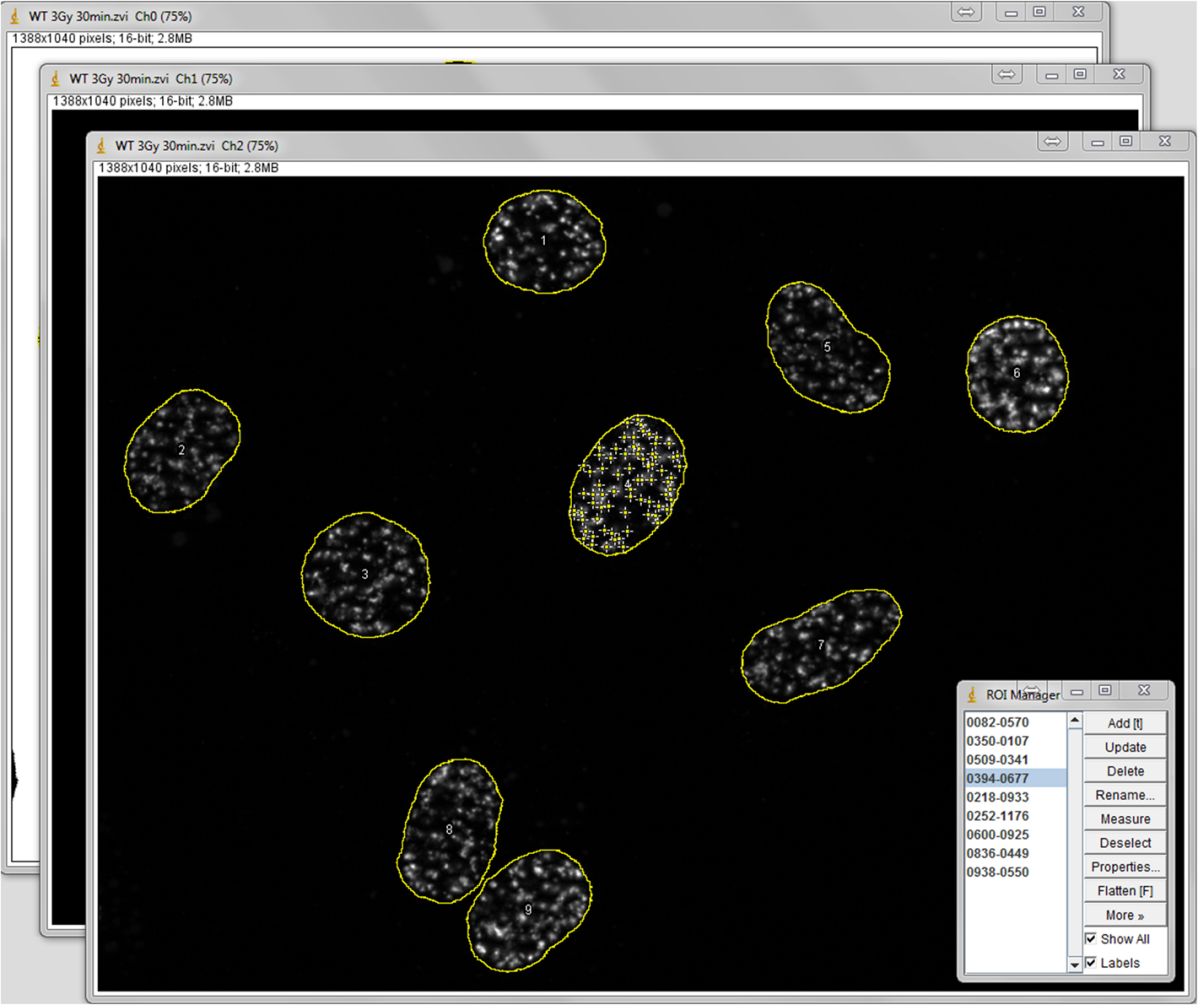
The Focinator counts foci for each pre-selected ROI automatically. Figure 3 demonstrates the calculation process stopped at the automated foci finding step for all ROIs. The image shows the selected foci in ROI 4. This part of the automation is based on the user’s noise level settings and on the previously marked ROIs, which are directly imported to the foci channel. Foci counting is followed by the closing of all channels and the immediate export into data files. The ROIs information will be imported into the export files in the order they were displayed in the ROI Manager window and named as numbers starting from one

To develop the Focinator as a tool for automated quantitative and qualitative analysis of foci with ImageJ, we first integrated a user-friendly interface. The interface (Fig. 1) includes eight buttons, a menu as well as nine shortcuts for the following commands < F1 > *Automated Mode*, <F2 > *Options*, <F3 > *Thresholding*, <F4 > S*eparation*, <F5 > S*electing ROIs*, <F6 > *Thresholding and Selecting ROIs*, <F7 > *Analyzing - Foci Count*, <F8 > *Open Next Image* in the folder. The menu also includes further information under *About The Focinator* and an instruction manual under *Help*. The second step for the development was an automated selection of the regions of interest (ROIs), such as cells or nuclei, depending on their appearance (Fig. 2). Moreover, automated detection of foci and the analysis of ROIs and foci were included (Fig. 3).

When running the Focinator, the selected ROIs are measured, including the area as well as mean, minimal and maximal grey values within the selection. The foci are detected based on the “Find maxima…” command of ImageJ. [24] Using the “Find maxima…” command, ImageJ identifies signal peaks of the 16-bit grey scale of an image compared to the grey scale values of the surrounding pixels. Testing of the Focinator was performed by capturing fluorescence images for detection of γH2AX foci. In this case higher fluorescence intensities of a putative focus correlate with an incensement of grey values in the image file. Importantly, the thresholds or contrasts of the foci images are not altered. The user might add a value for the noise level to the “Find maxima…” command, to disregard lower grey values caused by background noise. Background noise can be caused by unspecific staining due to unspecific antibody binding, insufficient blocking or washing. The maximal, mean and minimal densities as well as the localization and determination of ROIs size and intensity are also measured (Fig. 3).

The Focinator can either be run in an automated mode or in a semi-automated mode (shortcuts < F3 > to < F8>), respectively, with the possibility of manual addition or deletion of ROIs for better control and adjustments. After starting the *Automated Mode* via the button or < F1>, the macro selects the ROIs automatically using a preset threshold. When choosing “active separation”, it separates the cells. After this, the foci are counted and the results are saved in the chosen directory (Fig. 1.1 and 1.2). After having tested and adjusted the parameters on several pictures, it is possible to run a batch mode. We recommend testing parameters on multiple pictures before running the batch mode [10]. The batch mode analyzes all pictures in a selected folder including all subfolders. After completion of the batch analysis all retrieved values are summarized and means are calculated.

### Adjusting preferences in Focinator options

Preferences of the Focinator can be changed in *Options* < F2 > (Fig. 1.3). At first, the analysis mode has to be chosen, e.g. multi-channel analysis or separated pictures for each channel. The basic multi-channel analysis uses one channel for ROI selection and one or two foci channels, e.g. based on different stainings. The criteria for ROI selection can be defined in *ROI Settings,* a sub-paragraph of the *Options* window. The aforementioned window also includes preferences for the threshold level of the picture, the *size and circularity of included particles*, *separation* of overlapping cells or *exclusion of areas being cut by the frame*. For detection of foci a suitable *noise level* can be set. Finally, the last two dialogs of the options window offer the opportunity to change the saving directory and the file format.

For data export, we chose MS Excel because this program is widely used for spreadsheet calculation including the scientific context. Moreover, it enables further statistical analysis, presentation in graphs and charts, as well as easy export into other statistical software.

### Comparison of the Focinator to manual analysis and counting with ImageJ without automation

**Fig. 4:**
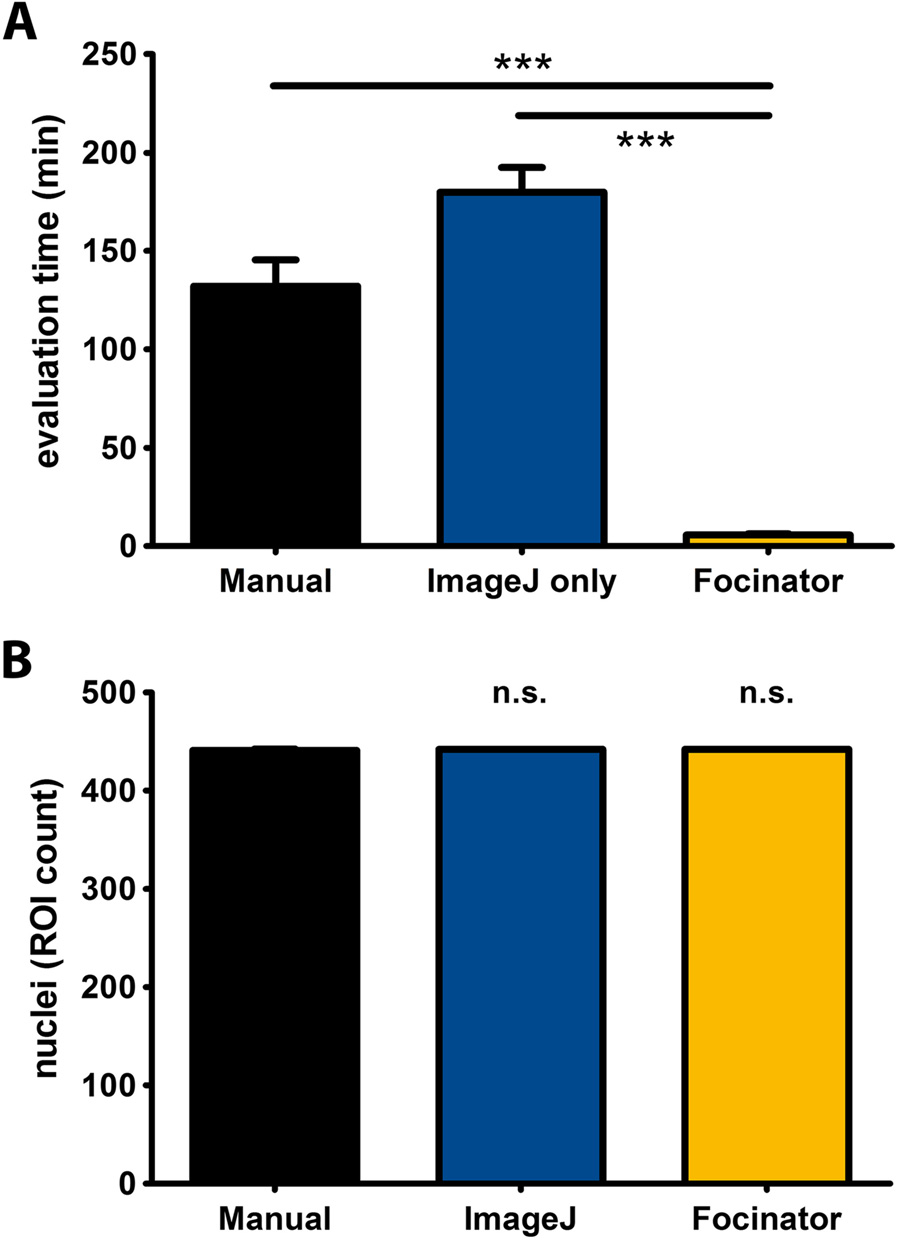
Use of the Focinator macro reduces counting times compared to ImageJ-based counting and manual evaluation. TRAMP-C1 cells were irradiated with 3 Gy. The cells were fixed and permeabilized for 15 min with 3 % PFA and 0.2 % Triton X-100 at different time points after irradiation. The nuclei were stained with Hoechst 33342. DSB foci were labeled with Alexa Fluor 647-linked anti- γ-H2.AX antibodies. The evaluation time for the same 35 multi-channel images containing 439 nuclei was compared between the analysis with the Focinator, ImageJ-based counting via manual ROI marking and “*Find Maxima…*” function or manual counting. **a** Evaluation times using the different counting methods. **b** Comparison of detected nuclei numbers by ImageJ-based analysis, Focinator batch mode and manual counting shown as overall ROI count

We tested the Focinator by counting radiation-induced γ-H2.AX foci in TRAMP-C1 cells at different time points after exposing the cells to 3 Gy. The results of the Focinator-analysis were compared to manual analysis as visual method and ImageJ-based counting via manual ROI marking and “Find Maxima” function as described by the Light Microscopy Core Facility -Duke University and Duke University Medical Center (Fig. 4) [24]. Manual counting of foci from images was chosen in the present study. By processing 35 multi-channel images, we counted 439 nuclei. Our software significantly reduced the analyzing time by a factor of approximately 23, from 132.07 ± 13.44 min for manual analysis to 5.61 ± 0.67 min with the Focinator (Fig. 4a). Surprisingly, evaluation with ImageJ without automation via macro needed more time than analysis with the Focinator or even the manual analysis (Fig. 4a). Nevertheless, analysis by ImageJ allowed the acquisition of more information about foci and nuclei than manual analysis. Importantly, there was no difference in nuclei detection between ImageJ-based methods and manual counting (Fig. 4b). Image acquisition was not part of the analyzing time; as fluorescent stainings are not stable, it is necessary to save image files for permanent documentation of the results with different counting methods, manual and automated. Moreover, image files can be used for more convenient manual foci counting with the option to mark counted foci with the software to avoid mistakes. Manual counting from images was chosen in the present study. In their routine protocol, Moquet *et al.* reported 1.5 h for counting non-irradiated cells and thus 4.68 s per cell to 6.1 h for irradiated cells and thus 19.06 s per cell for scoring of 20 cells in 96 samples. Thus, in comparison to Moquet *et al.* our manual scoring took about 59 min longer with an average of 30 s per cell. One explanation for our slower manual scoring is the higher radiation dose used - 3 Gy in our study compared to 0.5 to 1.0 Gy used by Moquet *et al.* and therefore a higher foci number per cell that amounted up to 70 foci per cell in our study compared to an average of 7 foci per cell in irradiated cells in the study of Moquet *et al*. Nevertheless, the Focinator would still be 13 times faster compared to the manual count of Moquet *et al*. [34].

The time for automated scoring in the Focinator’s batch mode and automated mode is mainly dependent on the computer performance. The batch mode can be executed in the user’s absence. Valente *et al*. reports about 30 s for loading setting parameters, the batch mode of the Focinator can be started after around 20 s. [19]. These data reveal that the new macro allows for highly accurate ROI selection, meaning that the nuclei or cells were selected in a valid and fast way. Moreover, we provide evidence that the Focinator excels manual analysis concerning time, effort for image processing and informative content, thereby corroborating with findings by others. However, the integration of an automatic ROI selection proved to be a valid and time-saving step, which is what makes the Focinator superior compared to other software solutions [13, 17, 20].

### Validation of the Focinator

**Fig. 5:**
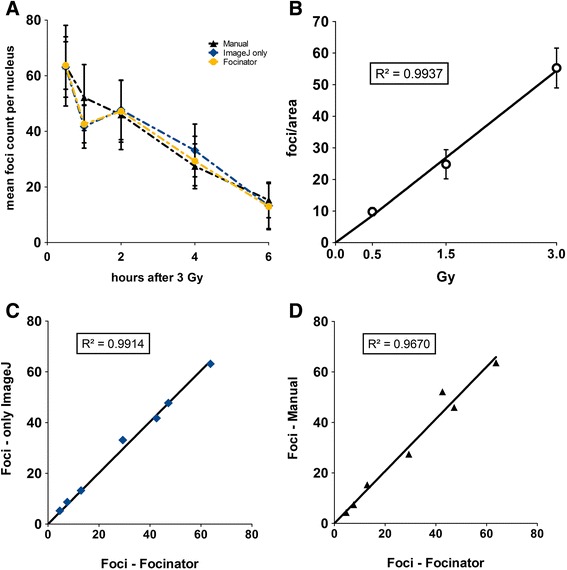
The Focinator’s accuracy is comparable to manual counting and evaluation only with ImageJ. ImageJ-based, manual counting and the usage of the Focinator macro were compared. To evaluate the repair time-dependent decrease of γ-H2.AX foci after irradiation TRAMP-C1 cells were irradiated with 3 Gy, incubated at 37 °C and fixed 0.5, 1, 2, 4, 6 and 24 h after irradiation. The cells were permeabilized and stained with an Alexa 647-linked anti- γ-H2.AX antibody. A total number of approximately 40 nuclei per time point was evaluated. **a** Development of the mean foci count per nucleus form three independent experiments at stated time points after irradiation. **b** A dose response curve depicts foci count after different doses (0.5, 1.5 and 3 Gy) 30 min after irradiation. A direct correlation between the different scoring methods with respective correlations value (R^2^) at the time points 0.5, 1, 2, 4, 6 and 24 h after irradiation is shown for Focinator-based evaluation in comparison to using ImageJ alone in (**c**) and compared to manual counting in (**d**)

For validation of the Focinator, TRAMP-C1 cells were irradiated with 3 Gy and foci were analyzed before and 0.5, 1, 2, 4, 6 and 24 h after irradiation. Again, the results of the Focinator were compared to ImageJ-based analysis and manual counting. All three methods showed a uniform time-dependent decrease of γ-H2.AX foci after the initial maximum at 30 min post-irradiation, proving the validity of the developed macro for reliable foci-counting (Fig. 5a). Counting of foci after irradiating cells with different doses (0.5, 1.5 and 3 Gy; 30 min after irradiation) was performed to validate the use of the Focinator at different amounts of DNA damage. The dose response curve shows a linear relationship between the number of foci per cell and the clinically relevant radiation doses used in the present study, thereby concurring with previously published literature [35–38]. Moreover, there was a strong similarity of foci numbers counted in 439 nuclei manually or with the Focinator (Fig. 5c, R^2^ = 0.9670). The comparison of ImageJ-based analysis with the Focinator achieved also high correlation indicating that the results of both analysis programs were very similar (Fig. 5d, R^2^ = 0.9914). Though a slight underestimation of counted foci was observed one hour after irradiation when using ImageJ and the Focinator compared to manual analysis, this effect was not significant. A similar phenomenon has previously been described by others and has been attributed to the increasing amount of overlapping foci at high foci numbers per nucleus, as well as at high irradiation doses and shorter repair times yielding increased foci size [12]. However, it has been suggested that the falsification of the results by high numbers of overlapping foci can be minimized when considering an additional analysis of foci intensity [25]. In contrast to manual analysis, the Focinator provides the opportunity to quantify the nuclei size as well as the minimal, mean and maximal intensity of the foci and the nuclei, and is thus superior to manual analysis. Another advantage of the Focinator is the opportunity to measure the ROIs area size. Accordingly, foci can be counted per area and not only per nucleus.

Our results support the conclusion that computational analysis is well suited to replace manual analysis with high accuracy; moreover it is time-saving and offers the opportunity to acquire further valuable parameters, such as nuclei size or the intensity of the foci. These values can be used for further normalization as explained in other publications [18, 25, 39]. In contrast, manual analysis is highly dependent on the experience of the investigator and requires extensive training [9–13]. Potential errors in manual analysis include multiple counting of single foci, counting of regions without foci and rare selection of less intense foci. [10] Finally, manual counting is not always reproducible [9–11]. Therefore, we and others recommend automated analysis to overcome the limitations of manual evaluation [9–13].

Moreover, manual analysis provides only a quantification of the number of foci, and there is no possibility of gathering additional information such as the size of nuclei or the intensity of the foci.

### Applicability of the Focinator for different users

**Fig. 6:**
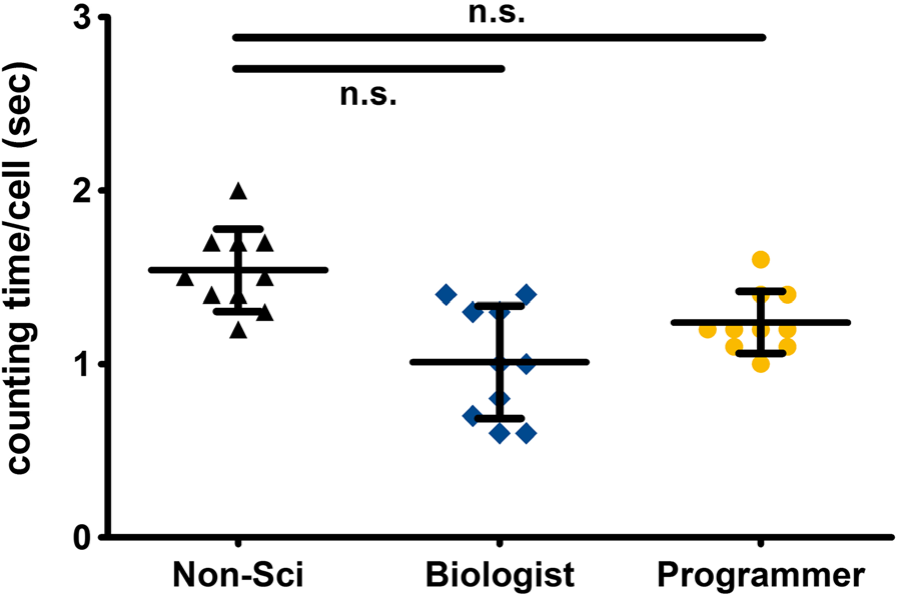
The Focinator is a user friendly method that can be used without long term training. In Fig. 6, three different groups of users are compared. Programmers of the Focinator (*n* = 2), Biologist (*n* = 2) and users with no scientific background (*n* = 2) evaluated ten different cell lines. For each cell line about 80 pictures containing a total of about 500 nuclei were evaluated by the different users with the Focinator. The graph shows the calculated evaluation times per nucleus including a correction based on the numbers of pictures that had to be opened

To prove the Focinator’s user-friendliness, the macro was tested by three different Focinator user groups, namely by the programmers of the Focinator (*n* = 2), by biologists (*n* = 2) and by users with no scientific background (*n* = 2) (Fig. 6). For the evaluation of applicability, all groups counted γ-H2.AX foci generated in response to different radiation doses in TRAMP-C1 cells at different time points post-irradiation using predefined parameters adjusted by an experienced scientist. In total 24,858 nuclei in 3361 images were counted. All users were able to use the Focinator after reading the software’s instruction manual. The data obtained by the programmers of the Focinator, the biologists and the users with no scientific background did not vary significantly in the mean evaluation times (Fig. 6). While the programmer needed 1.2 s per nucleus, the biologist needed 1.0, the users with no scientific background needed 1.54 s per nucleus (Fig. 6).

Users with no scientific background needed 21 min for counting 553 nuclei in 90 images in the first try. However, after the second analysis round, the untrained user needed only 14 min and 7 s for 653 nuclei in 80 images. The fastest analysis was executed in 10 min and 1 s for 461 nuclei in 82 images and was performed by the programmers. Although the measurements were performed by users with different professional backgrounds all investigators were able to successfully perform the analysis rapidly and particularly faster than by manual analysis.

The results obtained were rather similar, confirming the reproducibility of data. Evidently the Focinator achieves user-friendliness by redundancy of controls, like buttons and shortcuts, as well as by a menu and an implemented manual instruction as shown in Fig. 1.1 and 1.2.

Taken together, the Focinator is a user-friendly program. Moreover, a high comparability and consistency is achieved by automated computer analysis at increased velocity. This is achieved by automated analysis independent of the investigator’s prior knowledge if parameter setting is performed by an experienced researcher.

### Actual limitations of the Focinator and potential solutions

As outlined above, the Focinator is a valid open-source tool based on ImageJ for both the non-experienced and experienced user of scientific image processing alike. The Focinator offers advantages over manual analysis and already established software solutions. However, there are also limitations to its application. Since foci size and number varies depending on radiation dose and repair time, detection of overlapping foci can be difficult, a problem also recognized in manual analysis or when using an alternative software [12]. Because computational analysis offers measuring of qualitative parameters, a correction of these overlapping foci is possible by taking the intensity of foci into account. The Focinator counts foci based on signal intensity. This provides the opportunity to set a noise level to exclude foci with low intensity for further calculation, thereby strengthening the results [12, 25]. Cai and colleagues also suggested to include watershedding of foci to separate overlapping foci [25]. Watershedding is a procedure offered by ImageJ, which can be used for the segmentation of overlapping objects, like cells or even foci, in greyscale images [40]. We decided against this procedure, because watershedding of foci requires 8-bit formatting, which would cause the information about the signal intensity to be lost. Plugins such as 3D Object Counter [41], top-hat filter in Fast Filters [42] and FociPicker 3D [26] are alternative approaches to solving the problem of overlapping foci by taking size, intensities and algorithms into account. Though these options are more suited for the advanced user, implementation of the FociPicker 3D into the Focinator’s source code is feasible. Nevertheless, it is still possible that a single focus is too small to be detected with a confocal microscope, because the resolution of the microscope might be too low to display small foci separately [12, 43, 44]. To counter this problem, the macro offers the option of noise level adjustment. Lowering the noise level might result in higher foci numbers, due to noise artifacts been recognized as foci. The real number of foci can be validated by measuring the intensities of foci and taking these into consideration. As other authors have shown, not only the foci count, but also their intensity plays an important role and correlates well with the absorbed radiation dose. [12, 25] The use of an implemented cut off can further improve the results by deleting foci with a value below a chosen intensity to eliminate background signals. Intensities and XY-localization of each focus are exported into Excel. Setting a cut off for each channel and performing colocalization analyses are possible with these exported values.

Another limitation of the Focinator can be high cell density, which might result in overlapping cells and, thus, in overlapping nuclei. Therefore, it is not recommended to use overcrowded images. The result without use of watershedding or manual correction of the selection would combine two overlapping cells into one ROI with a larger area size. However, when using the Focinator the overall foci count will not be affected by a high cell density, because the foci count can be normalized by the area of the ROI.

However, it is not currently possible to analyze three dimensional or multilayer images with the Focinator/ImageJ while the IMARIS software (Bitplane AG) offers this possibility [13, 15].

Though being intuitive, the user needs to adjust parameters, such as the noise level, on their own, in contrast to FindFoci, where the program is able to learn the parameters on its own [10]. Although it is possible to perform analysis with predefined settings and an automated threshold, parameter setting for the individual cell type is a major step and can only be validly performed by people with sufficient background knowledge e.g. of the specific foci-related protein of interest. Parameters are supposed to be set according to image quality and the corresponding values found in published data such as 15–19 foci per nucleus per 1 Gy [35–38].

Another limitation of our macro is the restriction of multi-channel analysis to three channels only. This limits the macro to two foci channels with distinct fluorescence labeling (for example. using γ-H2.AX and 53BP1 antibodies with different secondary antibodies) since one channel is needed to select the ROI (e.g. using DAPI, 4′,6-diamidino-2-phenylindole to mark the nucleus).

### Advantages of Focinator-based foci evaluation

The Focinator is an inexpensive alternative to commercial packages. In contrast to the limited file formats accepted by some of the commercially available software solutions, the Focinator supports all file formats of ImageJ including TIFF, PNG, GIF, JPEG, BMP, DICOM and FITS, as well as raw formats. It is also possible to use stacked images and device-specific formats such as Zeiss’ AxioVision ZVI or Leica’s LIF [16].

The Focinator provides the possibility of adjusting multiple parameters for better image processing. Automated selection of cells or nuclei as ROIs is possible. For advanced users with prior knowledge of image processing who wish to adjust their analysis, the software offers further preferences and the choice of running the analyses in an automated mode or a semi-automated mode with the possibility of adding or deleting ROIs manually. It is even possible to analyze overlapping ROIs, a problem occurring in other automated solutions [13, 20].

Adjustability of the Focinator parameters is achieved without manipulating the images. The Focinator ImageJ macro exports the intensity and XY-localization of each focus. This step allows further processing of exported foci values, such as setting a cut off for each channel and colocalization. The raw data of the pictures are not changed for analysis by filters like changing of contrast, blurring or sharpening. This prevents the results from being manipulated [45]. We consider counting of one ROI per time and displaying the results as advantage and improvement in quality. Moreover, it is possible for the user to observe problems or incorrect preferences in selecting ROIs or foci counts. [13, 15] Being developed as a macro for ImageJ, the Focinator allows the implementation of new algorithms in order to customize the macro [14]. This is an important advantage of the Focinator compared to the standalone solutions. Due to the open-source nature of ImageJ, it is also possible with the macro to change the source code, to use the functions and plugins of ImageJ, and to program additional macros to solve the array of problems associated with image processing in a scientific context [13, 21, 22].

Further advantages compared to other established software solutions include the option to run the analysis in a batch mode and a data export into Microsoft Excel spreadsheet for further statistical and graphical evaluation. The Focinator Batch is programmed with R enabling modification by the user.

The possibility of observing the tool while selecting ROIs and foci in the batch mode, is very useful for further analysis security. This enables the user to recognize aberrant data and to adjust the settings accordingly.

## Conclusions

The Focinator is a costless, reliable and user-friendly open-source tool for fast automated high-throughput quantitative and qualitative analysis of DNA damage-induced foci formed by repair-associated proteins such as γ-H2.AX at the DNA damage sites with high accuracy and reproducibility. The Focinator is based on ImageJ with additional data export to Microsoft Excel. In comparison to manual analysis, it overcomes investigator-related bias and significantly reduces analyzing time. Moreover, it delivers a valid, fast and automated selection of nuclei and cells. Furthermore, it enhances the speed and reliability of analysis, and provides additional options for qualitative foci analysis like area size of nuclei and the intensity of foci. Importantly, the Focinator offers analysis of multi-channel pictures and colocalization. Its self-explanatory features make it possible to use the Focinator without prior training and the batch mode enables the user analyzing data in his absence. Data export into different output files with consecutive export into a spreadsheet is available, thus enabling further data processing and analysis. With the option to run data analysis in a batch mode, we think that the Focinator is a valid tool for efficient preclinical testing of the efficacy of new drugs targeting DNA repair alone and in combination with radio(chemo)therapy. For differing scientific aims, using further functions and plugins of ImageJ or programming of additional macros is possible.

## Additional file

Additional file 1:
**Supplement.** The Supplement includes a detailed description of the Focinator’s functions and commands. Moreover, it serves as an instruction describing installation, initialization and procedures.
