# Supplementary material for: The Focinator - a new open-source tool for high-throughput foci evaluation of DNA damage
Source: Radiat Oncol. 2015 Aug 4;10:163. doi: 10.1186/s13014-015-0453-1 (PMC4554354; doi:10.1186/s13014-015-0453-1)
Supplement: Additional file 1: — Supplement. The Supplement includes a detailed description of the Focinator’s functions and commands. Moreover, it serves as an instruction describing installation, initialization and procedures. [file 13014_2015_453_MOESM1_ESM.docx]

**Installation and initialization of the Focinator**

The Focinator can be executed with ImageJ as of version 1.47, and is applicable for either color or greyscale images in many different formats also including multi-channel files, like Zeiss ZVI or Leica LIF. To use the macro install the Focinator in ImageJ <Plugins < Macros < Install…>, copy the <Focinator_1.0.txt> file into the “macro” subfolder of ImageJ installation folder, or even better open <Plugins < Macros < Startup Macros… > delete the content and insert the whole text taken from the <Focinator_1.0.txt> file by copying it without line breakers. After restarting ImageJ, the new Icons of the Focinator appear. Several measurement parameters and thresholds can be adjusted for better automated detection of nuclei and foci. The macro consists of eight action tools, nine shortcuts and an additional menu. It gives the choice of running in an automated mode and a semi-automated mode with the possibility of adding or deleting ROIs manually for better control and adjustments as shown in Figure 1.

**Changing Preferences in Focinator Options**

The first implemented tool in the macro is an *options* action tool giving possibility of changing preferences. By using the red wrench button or via the shortcut <F2> the Focinator *Options* window is opened, offering the possibility of changing preferences for the analyses and adapt the macro’s behavior. (Figure 1.3) The *options* action tool provides a choice of preferences for *threshold*, *separation* of overlapping cells, *noise level*, *size of included particles, circularity of included particle*s. ROIs bordering the edge should be excluded. Moreover, changing the saving directory and the output format of the files is possible. The last preference to be choose is a decision between multi-channel analysis or just one channel analysis.

At first, the analysis mode has to be chosen, e.g. multi-channel analysis or separated pictures for each channel. The basic multi-channel analysis uses one channel for ROI selection, and one channel for measurement. It is also possible to count with two different foci channels in case of two separated foci fluorescence stainings that result in two different image channels. The stainings can be based on different antibody systems like anti-mouse and anti-rabbit or used in combination with antibodies directly linked to two different fluorescent molecules.

In a second step it is essential to choose the channel in which the foci shall be counted. The macro will automatically use the setting *1^st^ foci channel=front channel* for all pictures if not changed. If no second foci channel is used, the setting should be changed to *inactive*, meaning that the basic multi-channel analysis is used.

The next options that have to be defined are the *ROI Settings*. Depending on image quality, size, and magnification, it is recommended to *set the threshold* and the size filters for ROIs. The threshold level is adapted by setting the lower and upper limits for the threshold of contrasts or by using the *automatic threshold*, respectively. A preset standard threshold options is the *automatic threshold*. Settings should be chosen depending on the background intensity level. The *size of included particles* can be changed to a lower and upper value of pixels to ignore background noise or unspecific fragments, respectively. It is possible to adjust these values in case of larger or smaller objects or to exclude objects being cut by the picture frame. Overlapping ROIs might be separated by choosing “separate overlapping areas (watershed)“. The separation tool uses *smoothing, binarizing* and *watershedding* for separation of touching or overlapping ROIs. In case of the separation tool is not working properly, potentially because of thresholds being set wrongly or too many overlapping objects, it is possible to separate nuclei manually by drawing separating lines and filling these with the background color. If objects are not roundly shaped, it is possible to exclude those via *circularity* filters. A value of 1.0 indicates a perfect circle. As the value approaches 0.0 it indicates an increasingly elongated polygon.

The choice of the right *noise level* in the *Foci Settings* is an important step during evaluation.

Finally, the last two dialogs offer the possibility to change the *saving directory* and the *file format*.

**Selections of ROIs with the Focinator**

After starting the *automated mode* via the last button or <F1> as shortcut, the macro selects the ROIs using automatically a preset threshold. In case of choosing active separation, it separates the cells. After this, the foci are counted, and the results are saved in the chosen directory. For the automatic selection of ROIs, adjusting of the threshold is the first step of ROI selection. The ROIs are marked by signal intensity-triggered selection of pixels. This selection and ROI marking is based on ImageJ *Create Selection* algorithm with user options including filters for exclusion of ROIs bordering the edge of image, size minimum and maximum, watershed for overlapping objects and consideration of circularity. The automated selection of ROIs adds the ROIs to the ROI Manager (Region of Interest Manager). The selection is necessary for area specific counting of nuclei or single cells. The ROI selection also prepares area measurements and data export for single ROI values including foci count and intensity information like area, mean, maximum and minimum gray values. When the *automated mode* is not used, it is possible to select new areas and add these to the ROI manager, or delete unwanted elements. (Figure 2)

**Foci count with the Focinator**

The foci analysis consists of two procedures. In the first step the selected areas in the ROI list obtained in the selection step or selected manually are *measured*, including the area and mean, minimal, maximal grey value within the selection. The area describes the size of the selected ROIs in square pixels. The minimal and maximal grey values show the faintest and the strongest grey value. Moreover, the mean value describes the sum of all grey values in the selected ROIs divided by the number of selected pixels. In the next step, the number of foci per ROI is successively counted for each ROI using the ImageJ command *find maxima*. Particles outside the range of the set noise level are being ignored. This part of the automation is based on the user’s noise level settings and on the previously marked ROIs, which are automatically imported into the foci analysis channel. In addition to the count of foci, the maximal, mean and minimal densities are defined, as well as the localization and determination of ROIs size and intensity. (Figure 3) After this the list and mask of ROIs is being saved in a file named ROI.zip in your chosen directory. You can obtain the used ROI mask from this file for checking the selected areas or repeating the measurement of foci with different parameters. After analyzing a second time, the ROI.zip file will be overwritten and cannot be restored.

**Saving of results for further processing with the Focinator**

The focus counting is followed by the immediate export into the data files. Information about the ROIs will be imported into the export files in the order they were displayed in the ROI Manager window and named as numbers starting from one. The measurement results of the ROIs are saved in the ROI.zip file, the foci count of each ROI is saved in a file named “cell” with a number of each measured ROI. These results can be exported into XLS.

**Installation and Initialization of the batch mode**

For the batch mode is an additional macro available written with The R Project for Statistical Computing. R is obtainable at <http://www.r-project.org>. Installation of this is important for batch mode, cut off definition and analysis of colocalization.

**Troubleshooting and further adjustment with the Focinator**

If not all nuclei were recognized, the analysis should be either repeated step by step with different parameters, including changes in *threshold, particle size* and *circularity, noise level*, or ROIs added or deleted manually. Automated mode works well in case of images having the same colors and contrasts, the preferences being adjusted and with nuclei being well separated, having similar size and brightness. In case of overlapping cells, try the option *separation*, consequently *watershedding* is used*. Watershedding* is an ImageJ command for separating adjacent objects. It is possible to use each function separately, in a semi-automated mode. These options are recommended for searching the right parameters, if there are difficulties with analysis of the pictures or if you consider adding or deleting ROIs. Moreover, it allows the user to comprehend and edit the Focinator functions to solve different problems. For this mode, it is possible to activate each function of the Focinator with a button, in the menu or with a shortcut: <F1> *automated mode*, <F2> *options of the Focinator*, <F3> *thresholding*, < F4> *separation*, <F5> *selecting ROIs*, <F6> *thresholding and selecting ROIs*, <F7> *analyzing and foci count*, <F8> *opening the next image* in the folder. Additionally the menu offers information about programming of the Focinator in the menu tool *about the Focinator* and instruction manual by selecting *Help* (Figure 1.2).
